# The Loss of Raw Material Criticality: Implications of the Collapse of Saudi Arabian Oil Exports

**DOI:** 10.1007/s10272-021-1015-4

**Published:** 2021-12-11

**Authors:** Ulrich Blum, Jiarui Zhong

**Affiliations:** Chair for Political Economy, Martin-Luther-Universität Halle-Wittenberg, Friedemann-Bach-Platz 6, 06108 Halle (Saale), Germany

## Abstract

Raw material criticality has played an important role in geostrategic thinking, especially since the crisis surrounding the price and supply of rare earths at the beginning of the 2010s. However, once dependency and strategic importance grow too strong, substitution efforts will take place that could reduce or even eradicate the previous criticality. Critical resources rarely become obsolete very quickly. However, this could happen in the case of crude oil because climate policy is forcing defossilisation, but also because artificial scarcity is falling as a result of geostrategic rivalries that are causing oversupply. This article analyses this process and the possible consequences using Saudi Arabia as an example. The development of a green hydrogen industry has potential, but it should not be overestimated in view of the absorption capacity of the economy.

The two oil price crises in the 1970s as well as skyrocketing prices of rare earths ten years ago have revealed the dependence of the developed world on certain critical raw materials. When geostrategic aspects are added (especially those relating to national security), scarcity at the business level results in a vulnerability at the national level. Counter-strategies may include hoarding or, due to recent societal pressure for increased sustainability, a more intelligent design of materials, products or deconstruction possibilities that enable efficient recycling. Finally, substitution strategies may be considered for materials (copper against glass fibre in landline communication), technologies (movies against TV) or goods (personal visits against web conferences). These could turn critical resources into non-critical ones.[1]

* This publication was made possible under the joint research cooperation Center for Economics of Materials between the Institute for Microstructure of Materials of Fraunhofer Association and the Chair for Political Economy of Martin-Luther-University Halle-Wittenberg.

Resource-rich countries, overwhelmed by income from resource sales, have three options that are listed here in decreasing order of economic efficiency: (i) forced investment strategies that use income for modernising the economy in an attempt to prepare the country for a period in which income from resources falls, but which may overstress the adaptation capacities of societies; (ii) investment in funds in preparation for periods of declining sales; (iii) domestic use, which in most cases overstresses the absorption capacities of national economies and leads to price hikes in local goods, endogenous deindustrialisation and a revaluation of the currency, thus reducing national competitiveness. This follows transfer theory (Samuelson, 1964; Balassa, 1964) and is the basis of what is called the resource curse (Auty and Mikesell, 1993).[2]

The three strategies often appear as hybrids; however, dominant characteristics can be identified. For example, Shah Reza Pahlavi applied the first strategy in the 1960s and 1970s when trying to propel Iran into modernity.

1 For a survey, see Blum and Wehrspohn (2021).

2 Five hypotheses widely accepted in the resource curse literature explain how natural resource endowment might impede economic development (Frankel, 2012): high volatility of the commodity price; the crowding-out of manufacturing sectors by the natural resource sector; the tendency to develop an autocratic and oligarchic institution; the tendency to develop an anarchic institution; and the appreciation of local currency.

This failed because of inner opposition but left the mullahs with a formidable industry structure. Today, Norway commands one of the world's largest investment funds. The Netherlands, on the other hand, spent gas income on domestic programmes that led to price hikes followed by wage increases in the non-tradable sector that subsequently spilled over into manufacturing. The loss of competitiveness drove the rail, car and aircraft industries out of the markets. It is known as the Dutch disease or the East German disease because of the similar effects of the stabilisation transfers from West Germany after unification (Blum, 2008).

It is clear that the loss of criticality will hit a type iii country hardest, and even if type ii funds are available, they may be syphoned off. Moreover, this may completely reverse financial flows in large countries and have a devastating effect on international financial stability.

Oil has been a critical resource for a long time; however, this is beginning to change. We use the Kingdom of Saudi Arabia, a major oil producer, as a reference. The country is in danger of economic collapse if oil prices suddenly fall – which is not beyond the realm of possibility.

The remainder of the article is organised as follows: In the next section, we analyse the roots of crises from a resource perspective. Using oil as an example, we point to the problem of managing financial inflows efficiently and to the impact of geostrategic rivalry. We then concentrate on two major factors that have led to a regime change in the balance between global oil production and consumption: fracking and defossilisation.[3] We subsequently apply this to Saudi Arabia and point to the medium- to long-term challenges for global stability given this regime change. Finally, we conclude that a substantial turnaround in investment management is required of (Middle Eastern) oil-producing countries to guarantee global stability.

## Geostrategic environment, criticality and crisis

Cheap oil has promoted the rapid industrialisation of the Western world and benefited oil-exporting countries by providing a sufficient influx of oil money, especially to those that joined forces and co-founded the Organization of the Petroleum Exporting Countries (OPEC) to set oil prices.[4] Many of these oil-rich countries have achieved oil-driven economic growth and joined the group of middle- and high-income countries.

Oil has been a critical energy resource since the 19th century. In 2018, crude oil made up 33% of the world's annual primary energy consumption (BP Energy, 2020). Since its wide industrial use from the late 19th century onwards, partly due to Rockefeller's efforts to monopolise the oil market and then the antitrust-led breakup of the Standard Oil Company, global oil consumption and production have been steadily increasing. In the 21st century, soaring demand in emerging economies has compensated for the stagnating oil demand from the developed world.

Today, oil still has a relatively low degree of substitution, particularly with respect to technology (transport sector, chemicals) and transport (large tankers, pipelines). This guarantees a stable demand despite volatile prices. Empirical studies support the relatively low price elasticity of the global oil demand, especially in the short run (Cooper, 2003; Caldara et al., 2016). Due to its low substitutability and the oligopolistic market structure, oil has provided its suppliers with a steady stream of revenues.

Having experienced an ever-increasing demand for fossil fuel, the second half of the 20th century was besieged by the economic threat of "peak oil". However, this concern about a negative supply shock has now mainly shifted to "peak demand". According to recent predictions, oil's dominance, and therefore criticality, will decrease rapidly in the coming 30 years. The main driver of this profound shift in mindset is environmental concerns about greenhouse gas emissions. In addition, fracking turned the US from an importer to an exporter of oil and gas. This has not only put pressure on prices, it decoupled the US strategically from the Middle East as a military safeguard of global oil supplies. In response, Saudi Arabia launched an oil price offensive, trying to drive the US fracturing industry from the market and indirectly hitting its biggest regional rival, Iran, in its strategy to modernise its industries. In fact, Saudi Arabia had switched from swing pricing, which tries to equilibrate oil prices to levels that satisfy both producers and consumers, to limit pricing.

3 We prefer to speak of defossilisation instead of decarbonisation because the principal momentum must be to limit the emission of fossil carbon.

4 The OPEC cartel was at the centre of a veritable economic war in the 1960s. It was founded in 1960 to achieve a lasting monopolistic position. During the Yom Kippur War, there was a supply boycott against the Western states to support the Arab states' attack on Israel. It has lost a considerable degree of influence through resignations and an increasing supply by many unaffiliated countries and the support of the Saudi regime by the United States, which tried to moderate prices for a long time.

Figure 1

**Development of crude oil prices, 1900-2018**

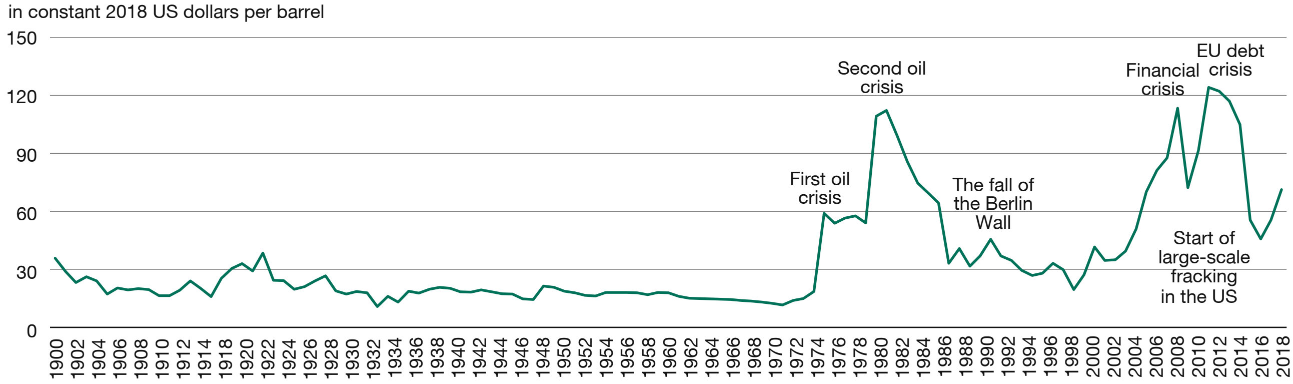

Notes: 1900-1944: US average; 1945-1983: the posted price of Saudi Arabian Oil F.O.B Ras Tanura; 1984-2010: Dated Brent.

Source: Authors' own illustration based on Nasdaq Data Link Crude, oil price from 1981, 2021.

Figure 1 shows the oil price development from 1970 to 2018. One of the first slumps started in the years before the Berlin Wall came down. It accelerated the breakup of the Soviet Union, leaving the US as the sole superpower.[5] In the summer of 2015, the Iranian nuclear treaty drove up the global crude oil supply, which, after 30 years of boycotts and sanctions, opened up Iran's access to the world market. Later, the US withdrawal from this treaty in 2018 again led to tensions on global oil markets; in addition, the US extraterritorial enforcement of sanctions on trading partners has also put pressure on the global economy.

The apparent oil price conflict described above is not only a multipolar economic war (Blum, 2020), it is also a hybrid military war in which economic rivalry serves as an essential and embedded driver. US fracking has not only reduced the scarcity on the international oil market, it has also reminded the traditional supplier countries of the finiteness of their competitive position, something which defossilisation is further eroding. Gundersen (2020), for instance, calculates that the US import shock, reflecting its domestic shale oil supply shock, accounts for 13% of the global oil price variation from 2003 to 2015, while OPEC explains about 17% of this variation. Saudi limit pricing led to a technology drive that more than halved initial fracking costs from over US $80 per barrel to about US $30 per barrel.

5 Studies supporting the resource curse include Sachs and Warner (1995), Collier and Goderis (2008), Sala-i-Martin and Subramanian (2013), and Dartey-Baah et al. (2012). Studies rejecting the resource curse include Stijns (2005), Brunnschweiler and Bulte (2008), Alexeev and Conrad (2011), Michaels (2011), James (2015). Welcome institutional reforms could, in fact, help turn the resource curse into a resource blessing (Humphreys et al., 2007).

## Defossilisation and oil's path towards non-criticality

Since the 19th century, the temperature of the Earth has risen by 1.14 degrees Celsius, resulting in observable negative impacts on its ecosystems. Climatologists today can identify a highly likely (95%) causality between human activity and global warming (IPCC, 2014). If carbon dioxid net emissions do not decrease, further warming will cause irreversible damage to the environment. Compared to all anthropogenic greenhouse gas sources, fossil fuel combustion and industrial processes accounted for about 78% of total emissions from 1970 to 2010 (IPCC, 2014). Therefore, the necessity of limiting global warming through defossilisation gained political and social acceptance and led to the 2016 Paris Agreement. Its ultimate goal is to keep the rise in global temperatures in this century to well below two degrees Celsius above the pre-industrial level or even below 1.5 degrees Celsius. To meet this goal, the entire world must achieve zero net carbon emissions by around 2065, with the odds of reaching the target exceeding 66% (Rogelj et al., 2015). Recently, the German Constitutional Court ruled that government implementation programmes must reflect the environmental positions of younger generations, thus pressing for more realistic reduction plans (BVerfG, 2021). A district court in The Hague ruled that Shell must drastically cut its greenhouse gas emissions because international climate agreements are also binding to enterprises. All of this will put additional pressure on oil prices.

From another angle, the projected expansion of renewables mirrors a potentially sharp reduction in the market share of oil. During 2018 and 2020, renewable energy production grew at an annual rate of 6%. If the climate targets are met by 2050, the share of renewables in primary en-

Figure 2

**Production of gasoline and other liquid derivatives**

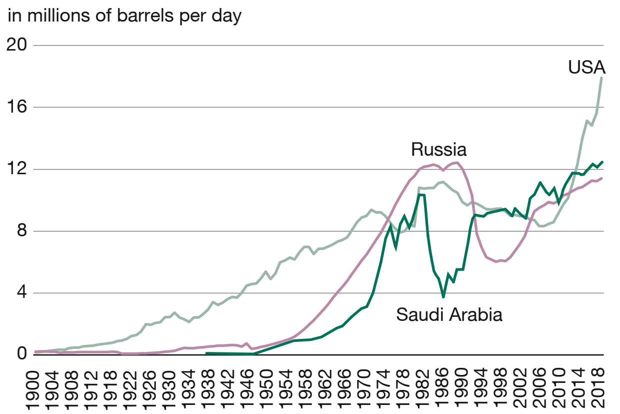

Sources: Etemadd & Luciani (1900-1980) and US EIA Historical Statistics (1981-2018), own illustration.

Figure 3

**Saudi Arabia's oil vulnerability, 2000-2019**

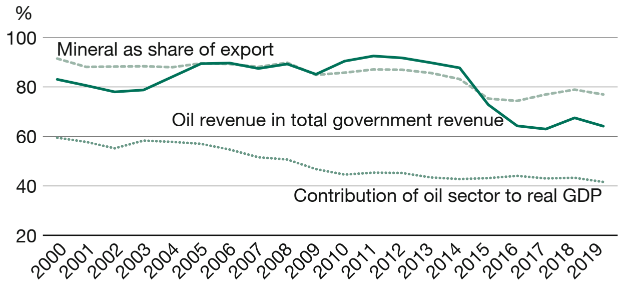

Source: Saudi Arabian Monetary Authority, own illustration.

Figure 4

**Saudi Arabia's GDP by sector, 1970-2018**

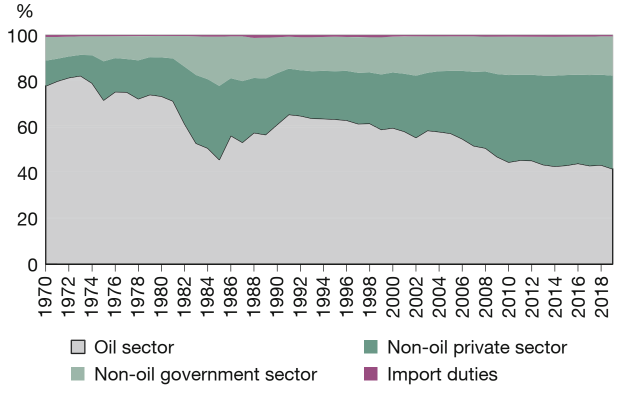

Source: Saudi Arabian Monetary Authority, own illustration.

ergy would increase from 4.7% to a value between 44.3% and 59.2% according to BP's Energy Outlook (2020). It further forecasts a 55% and 80% fall in oil demand in two intense decarbonisation scenarios, meeting the target of two and 1.5 degrees Celsius, respectively.[6] The report also conjectured that oil demand peaked already in 2018; henceforth, global oil demand would trend downward. Following the World Energy Outlook (IEA, 2020), its "net zero emissions by 2050" would lead to a fall in the present value of the world's oil sector by almost 50% by 2040 compared to 2019, and pre-COVID-19 consumption patterns would not be reached before 2023. Shocks may drive up the price, e.g. as OPEC+ chooses to curb supply or the Houthis attack Saudi facilities. However, for many oil-producing countries, oil prices may just oscillate around their fiscal breakeven oil prices (IMF, 2020).

### Vulnerability and the Saudi Arabia's position

Saudi Arabia is typical of a country that has earned its national wealth from crude oil reserves. In the first decade of the new millennium, Saudi Arabia was the number one global oil supplier. Later, the United States overtook it and Russia with its fracking revolution. This is depicted in Figure 2. As a result, despite the absolute increase in its oil production, Saudi Arabia witnessed a shrinkage of its global oil share.

Saudi Arabia's oil revenues are substantial relative to its economic size, as different indicators in Figure 3 document. In the first two decades of the 21st century, the oil sector accounted for around 40% of Saudi Arabia's total GDP and contributed to about three-quarters of its governmental revenues. In 2019, oil revenues made up about 64.1% of its government's annual revenues, and the oil sector contributed over 40% to Saudi Arabia's real GDP. As a relatively small economy with little industrial diversification, Saudi Arabia has to sell most of its oil reserves to the rest of the world and is therefore intensively engaged in the oil trade. In the new millennium, Saudi Arabia's export-to-GDP ratio has averaged about 40%, 85% of which are mineral exports.

The high importance of oil in the composition of Saudi Arabia's GDP is also depicted in Figure 4. It shows the enduring problem of phasing out oil dependency, which is difficult because oil plays a major in fiscal stability. The IMF estimates that Saudi Arabia's external and fiscal breakeven oil prices in 2020 are US $45.3 and US $66 per barrel, respectively. Given the long-term price pro-

6 In absolute terms, oil consumption would decrease from 190 EJ to about 89 EJ and 42 EJ in the two scenarios.

Figure 5
**Specialisation pattern of Saudi Arabia, 2018**

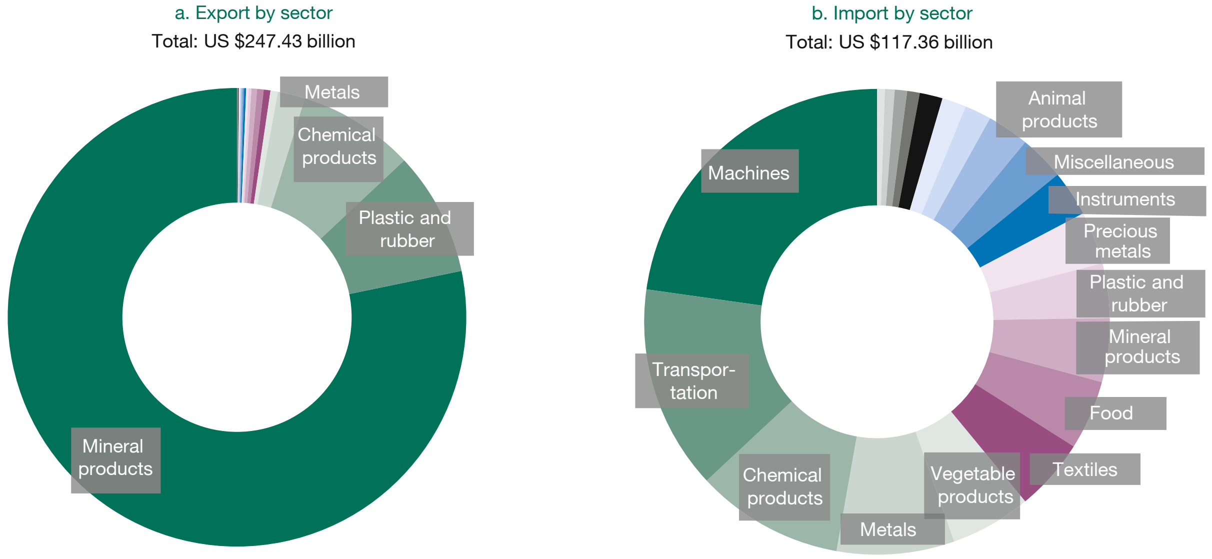

Source: Simoes et al. (2011), own illustration.

jections, a depletion of Saudi Arabia's investment funds starts to become a reality.

Figure 5 shows Saudi Arabia's import and export structures in 2018 and is proof of an oil and oil-derivative dependent industry structure that finances a highly diversified import basket. In brief, Saudi Arabia is a highly specialised Ricardian country that thrives on its comparative advantage: oil. It exports resources in exchange for products that are knowledge, capital and labour intensive. Intra-industry trade, which is often observed in countries actively participating in global value chains (GVCs), is less discernible.

By producing first blue and later green hydrogen, Saudi Arabia could try to set up a new pillar for exports and use crude oil only as a material base for carbon-based products, e.g. plastics. Over and above the limitations already apparent in the export portfolio of oil-derivative commodities (Figure 5a), limits may emerge from the ability to absorb the necessary investments and geostrategic reliability. In fact, assuming a price of about US $1.5 per kg, exports would have to amount to some 128.67 billion kg.[7] More is needed if other specialised products should replace present exports like rubber or basic chemicals, such as ammonia. Investments in the necessary hydrogen production facility would amount to US $1,400 billion.

7 The data used here are taken from Dii Desert Energy and Roland Berger (2021), Fasihi and Breyer (2019), PwC (2021), Yates et al. (2021).

At least another US $400 billion should be added for the other third of export production, i.e. green steal, ammonia (as currently planned) or other chemicals. Over a period of 20 years, this would imply annual capital expenditures of some US $90 billion. They should be larger in the early years, i.e. the 2020s, and decline later. As Saudi Arabia would not be able to produce the necessary investment goods and supply the necessary labour, the current account balance would come under stress unless most of the investments are FDI financed. If historic budget surpluses and, thus, investment into overseas funds are reversed, the resulting impact on financial stability and on exports from manufacturing countries could have the potential to trigger a crisis.

## Saudi Arabia's position in the global value chain

Intra-industry trade is one of the newly discovered stylised facts in the international trade pattern, particularly after the 1980s. It contradicts the premises of traditional trade theory (Inomata and Taglioni, 2019). In contrast to the Ricardian Saudi Arabia, the world economy has evolved into a brand new stage of global production fragmentation. This has allowed many economies, especially in Asia, to escape the poverty trap and develop a broad industry base – unlike Saudi Arabia.

A cross-country GVC analysis allows us to locate Saudi Arabia in the global production network and calculate the forward and backward production length of Saudi Arabia and other selected countries based on the methodology of Wang et al. (2017) and the OECD Inter-Country Input-Output (ICIO) data. We use the ratio of the forward and backward production length index to calculate Saudi Arabia's relative position in global value chains for 2005 and 2014 and show the dynamics.

Figure 6 demonstrates the results. The x-axis and y-axis represent backward and forward production lengths, respectively. In this representation, the dots in the bottom left corner represent countries with the shortest average production chain lengths; as a dot moves towards the upper right, the total production chain it represents gets longer. From the other angle, dots in the bottom right represent countries positioned further downstream in the GVCs, with short forward linkages and long backward linkages. As a dot moves towards the upper left, the country it represents moves forward along the GVCs. This way of mapping forward and backward linkage length and GVC position is credited to Escaith and Inomata (2016).

As Figure 6 suggests, Saudi Arabia has an overall short GVC length and is positioned at the very upstream end. In 2014, its average forward and backward indices were 2.11 and 1.54. In the same year, the world's forward and backward indices are equal at 2.02. A shorter production chain length demonstrates less vertical specialisation on the sector level in the first approximation, indicating less industrial network complexity. Usually, the phenomenon of an increasing complexity of the production network accompanies industrial development. In this sense, the lower index can be explained by Saudi Arabia's weak industrial base, as its industrial complexity lies below the world average. When comparing 2014 to 2005, Saudi Arabia's total production length has dropped from 3.74 to 3.65, whereas the world average has increased from 3.90 to 4.04. Therefore, while the world has averaged a deeper vertical specialisation, Saudi Arabia has moved against this trend. In terms of its GVC position, Saudi Arabia is positioned far upstream, with no remarkable change from 2005 to 2014. This slight change corroborates the country's slow progress in shifting away from oil to a more industrialised economy.

In stark contrast, Asian countries, hotspots for global investment in recent decades, gather in the upper right quadrant. They share the common features of relatively long production chain lengths and a downstream GVC location. Due to the resource curse described above, Saudi Arabia's relatively high labour costs factor into its limited GVC participation. Without a substantial increase in labour productivity or a declining exchange rate, it is difficult for Saudi Arabia to compete for FDI against emerging economies with more favourable labour costs.

Figure 6
**GVC analysis of Saudi Arabia and selected countries**

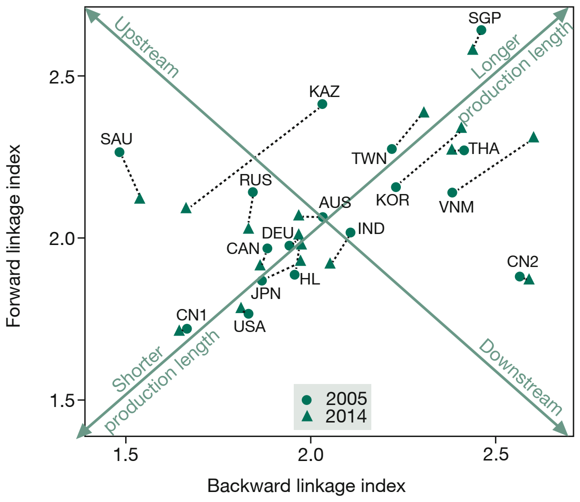

Note: Data are presented for 64 countries (i.e. 36 OECD countries and 28 non-OECD economies), the rest of the world, and split tables for China and Mexico. Data on Mexico and China are split into MX1, MX2, and CN1, CN2, respectively. CN1 represents China's activities, excluding export processing; and CN2 represents its export processing activities. In the same vein, MX1 and MX2 represent Mexico's activities (excluding export processing) and its export processing activities, respectively.

Source: Authors' own calculations.

Investments in a hydrogen economy would most likely not change this basic pattern of international labour division.

## Dutch disease as a factor in limiting Saudi Arabia's economic growth

Oil revenues have had an overall positive impact on Saudi Arabia's real GDP per capita (Alkhathlan, 2013). Anser et al. (2020) also conclude that Saudi Arabia's oil rent looked in fact like a "resource blessing". The reason is that, given its small population, the Kingdom has sufficient opportunities to successfully invest in industrialisation for the benefit of its entire population. From 1990 to 2014, Saudi Arabia experienced a year-on-year average growth rate of 9.35% on its industrialisation intensity index. However, its industrialisation process still lags behind emerging East Asian countries such as Vietnam, Indonesia and Thailand (UNIDO, 2020).

However, Dutch disease selectively hit Saudi Arabia after the oil boom (Looney, 1990; Parvin and Dezhbakhsh, 1988; Ahmad Bajwa et al., 2019). The Saudi riyal's real effective exchange rate remained appreciated for most of the 1970s. This made exporting expensive and importing cheap (Looney, 1990; Ahmad Bajwa et al., 2019), reduced industrial output, especially in major non-oil sectors

(Looney, 1990), increased local labour costs and impeded industrialisation. As a result, migrant workers constituted over two-thirds of the country's total labour market from 2005 to 2015. An investment bonanza into first blue and later green hydrogen in competition with other countries may, given the above-mentioned composition of the economy, overburden the absorptive capacity. We may take the transformation of East Germany as a reference case, as in both cases, a new economy had or has to be (re-)capitalised.[8] Compared to Saudi Arabia, it had 50% of the population and 60% of employment. Annual investments averaged 60 billion euros in the 1990s (30% plant and equipment, 50% housing, 20% infrastructure) and led to severe absorption problems. Assuming that, over and above the industrial investments, Saudi Arabia would also need investments into housing and infrastructure, severe macroeconomic adaptation problems will surely arise – from a revaluation of the currency to price hikes for local goods and the destruction of existing industries.

Countries with relatively weak industrial bases could in fact improve their productivity, know-how and export diversification by participating in the GVCs (Kowalski et al., 2015). However, the Dutch disease might have deprived the Saudis of their opportunity to adopt such an economic development strategy.

### Challenges facing Saudi Arabia's economic transformation

Against the backdrop of the phasing out of oil, the Kingdom must decouple its economy from the potential downside risk of oil prices. In 2016, it adopted the most rigorous transformation strategy since the 1970s, the Saudi Vision 2030 (2016), aiming to invest in a more diversified and industrialised economy and ending its oil dependency before fading oil revenues exhaust its ability to finance domestic projects in the industrial, mining, energy and logistics sectors.

However, Vision 2030 faces political and economic challenges for rent-seeking reasons, especially interlocking interests and institutions opposing a decoupling from oil. Even if the recent oil price slump reduced such dissenting voices, they would always come back as soon as the oil price recovers and obstruct the transition (Moshashai et al., 2020). Figure 7 shows the dependency of the Saudi Arabia's GDP on oil. Oil prices have impacted the Iranian revolution, the Iran-Iraq war, the Gulf Wars and the global financial crisis of 2009. The oil price shocks in 2014 and now the COVID-19 pandemic have significantly hurt Saudi Arabia's real economy (Jawadi and Ftiti, 2019). According

8 Data is taken from Blum et al. (2009a) and Blum et al. (2009b).

Figure 7

**Saudi Arabia's GDP and oil prices, 1970-2019**

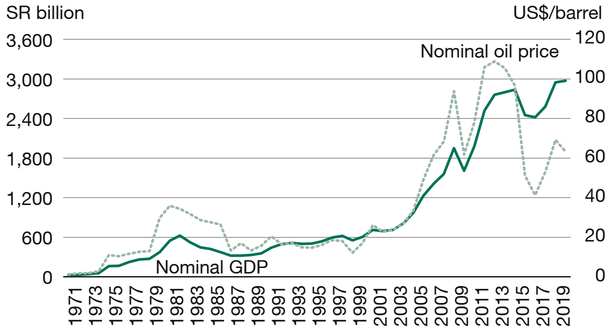

Sources: SAMA; and World Bank Commodity Price Data, own illustration.

to the IMF's estimation, the Saudi government's budget deficit for 2020 is estimated to be 12% of its GDP.

Furthermore, to finance the Saudi Vision 2030, the government announced a doubling down on oil production before the resource becomes valueless. Such a policy is in line with the green paradox hypothesis because the future lack of demand would generate even worse revenues (Sinn, 2008a; 2008b). However, overwhelming oil revenues are likely to come with the risk of being infected by the Dutch disease, which might jeopardise Saudi Arabia's diversification efforts and leave the Kingdom with an even stronger dependency on oil.

Several empirical studies have confirmed the close nexus of oil prices and Saudi Arabia's real economy. Algahtani (2016) uses VAR and VECM models to verify a significant and positive long-run relationship between oil prices and Saudi Arabia's GDP between 1970 and 2015. Aloui et al. (2018) use wavelet methodology to demonstrate strong but non-homogenous linkages between oil prices, riyal/US dollar exchange rates and real GDP. Jawadi and Ftiti (2019) identify nonlinearities and threshold effects. By regressing GDP and government revenues (per capita) to the oil price, Vandyck et al. (2018) estimate that the elasticities of the GDP and government revenues with respect to oil prices are 0.5 and 0.9, respectively. Using the estimation by Vandyck et al. (2018), a 30% fall in oil prices would cause Saudi GDP to contract by 15%, other things being equal. This rough calculation illuminates the vulnerability of the Saudi economy. And the prospects are bleak: Although financial reserves are high, financial markets have started to punish the country for its industry structure and oil dependency, which became visible when the IPO of Saudi-Aramco failed. Only a wise, import-led investment strategy into one's own diversified economic base could mitigate or even reverse such a trend and raise productivity (Adam and Bevan, 2006). Furthermore, such a strategy might overcome the highly probable risk of a J-curve effect if a strong devaluation occurs as a result of an oil price crisis.

## Policy outlook

A stabilisation strategy for the Saudi economy would have to harmonise the expected depletion of oil income, create the necessity to diversify its economy in time, and exploit locational advantages and new technologies in order to guarantee long-term competitiveness. From a global perspective, such a strategy should aim at stabilising, not disrupting world financial flows.

A future may emerge from the hydrogen economy: A transition path could start with blue (fossil-based) hydrogen and switch to green hydrogen based on solar and wind power alongside the development of a new “carbon culture”. One principal export good would be hydrogen or methane, encompassing all the high-end research and industry functions that go with it and a “jump” into a new innovation cycle.[9] However, the required investments must be accelerated in order to offset possible declines in oil demand, which may run against efficiency and effectiveness and may be delayed by counter-effects through limits of economic absorption.

The same logic could apply to other oil-dependent countries with similar economic structures. In this case, the decriticalisation of oil would be as catastrophic for other oil-dependent regions as it would be for Saudi Arabia. The collapse of the exchange rate and currencies, such as the Saudi riyal, and the contamination of other oil-exporting countries’ currencies might eventually threaten the stability of the highly interconnected global financial system.

9 In the case of Germany, the phasing out of atomic and coal energy may result in blackouts as the necessary electrification of the economy lags behind. To supply the Bavarian industry, the power link currently being built across the Bavarian border to Thuringia would have to be quadrupled, which is politically unfeasible. However, the existing gas grid and the newly built hydrogen grid could be perfect substitutes to supply existing or newly constructed, decentralised power plants.
